# Intraoperative dexmedetomidine administration and acute kidney injury in patients undergoing unilateral partial nephrectomy: a retrospective study

**DOI:** 10.1080/0886022X.2024.2409334

**Published:** 2024-10-01

**Authors:** Ryan Wong, Juan Jose Guerra-Londono, Arun Muthukumar, Nicolas Cortes-Mejia, Diana Fernanda Bejarano-Ramirez, Juan Pablo Cata

**Affiliations:** a Tilman J. Fertitta Family College of Medicine, University of Houston, Houston, Texas, USA; b Department of Anesthesiology and Perioperative Medicine, The University of Texas MD Anderson Cancer Center, Houston, Texas, USA; c Anesthesiology and Surgical Oncology Research Group, Houston, Texas, USA; d Department of Pain Medicine, The University of Texas MD Anderson Cancer Center, Houston, Texas, USA; e Department of Transplantation and Hepatobiliary Surgery, Bogotá, Colombia

**Keywords:** Dexmedetomidine, acute kidney injury, partial nephrectomy, renal cancer

## Abstract

Partial nephrectomies are associated with an increased risk of acute kidney injury (AKI), but dexmedetomidine administration may improve renal outcomes. We hypothesized that intraoperative dexmedetomidine administration would be associated with a decrease in AKI development in patients undergoing unilateral partial nephrectomy. In this retrospective study, adult patients who underwent unilateral partial nephrectomy from April 2016 to October 2023 were included. Exclusion criteria were a history of end-stage renal disease, ineligible procedures (i.e., aborted procedure, conversion to radical nephrectomy, surgery on a horseshoe kidney), and reoperation within three days of the initial nephrectomy. Patients were categorized according to whether they received intraoperative dexmedetomidine. The primary outcome was AKI incidence within three days of surgery; AKI was defined according to the Kidney Disease Improving Global Outcomes definition. Propensity score matching (PSM) was conducted to account for potential confounders (age, body mass index, sex, American Society of Anesthesiologists score, final surgical approach, clamping-related ischemia for >15 min). We included 1,632 patients; 214 received dexmedetomidine and 1,418 did not. Before PSM, the AKI rate was 31.2% in patients who received dexmedetomidine and 25.7% in patients who did not (*p* = 0.081). After PSM, the AKI rate was 31.3% in patients who received dexmedetomidine and 27.6% in those who did not (*p* = 0.396). The post-PSM odds ratio for AKI following dexmedetomidine administration during unilateral partial nephrectomy was 0.910 (95% CI: 0.585–1.142; *p* = 0.677). Intraoperative dexmedetomidine was not associated with a reduction in postoperative AKI incidence or severity after unilateral partial nephrectomy.

## Introduction

The incidence of kidney cancer is increasing in the United States; the American Cancer Society estimates that 81,800 new cases involving the kidney and renal pelvis were diagnosed in 2023. Treatment guidelines for renal cell carcinoma, the most common solid tumor in the kidney, have evolved over time, and advances in imaging and surgical techniques now enable earlier detection and more selective tumor dissection with localized partial nephrectomy than with radical resection [1,2]. Furthermore, clinical guidelines recommend partial nephrectomy for the treatment of early-stage renal cell carcinoma when feasible to minimize the risk of chronic kidney disease [1,3–5].

Although partial nephrectomy is mostly used to preserve maximal renal function, surgical injury resulting from vascular clamping–related ischemia and functional parenchymal excision is known to increase the risk of perioperative acute kidney injury (AKI) and subsequent functional loss [6,7]. Studies have reported AKI incidence rates between 14.4% and 41.8% after partial nephrectomy, but Zhang et al. reported a 54% AKI incidence rate in patients with a solitary kidney [6,8–10]. Worse renal function has been associated with significant morbidity and mortality [6,11]. Numerous modalities (e.g., loop diuretics, renal-dose dopamine, mannitol) have been proposed to prevent or manage perioperative AKI, including dexmedetomidine [12–15].

Dexmedetomidine is a highly specific α-2 adrenoceptor agonist used in surgery as an adjuvant anesthetic [16,17]. Over the last decade, studies in murine models have suggested that dexmedetomidine administration, before or after a renal ischemia/reperfusion injury, acts *via* several anti-inflammatory pathways to significantly reduce the levels of inflammatory mediators and oxidative stress markers while decreasing the levels of serum creatinine and improving histologic markers of renal function [18–22]. Gu et al. investigated renal ischemia/reperfusion injury in mice and showed that dexmedetomidine conditioning reduced histopathologic kidney damage and levels of plasma inflammatory markers and increased survival rates 7 days after the procedure [23]. A meta-analysis showed that dexmedetomidine protected against AKI in a large cohort of patients who underwent cardiac and non-cardiac surgery [24]. In a randomized controlled trial, a low dose of (0.6 mg/kg^−1^) of dexmedetomidine given after renal artery unclampling significantly reduced the levels of biomarkers associated with early AKI and induced marginal gains in the glomerular filtration rate compared with placebo 1 month after laparoscopic partial nephrectomy [15]. However, no study has investigated whether pre-ischemic administration of dexmedetomidine impacts AKI after partial nephrectomies.

In the present study, we aimed to determine the association between intraoperative dexmedetomidine administration and AKI after unilateral partial nephrectomy. We hypothesized that intraoperative dexmedetomidine administration as anesthetic adjunct would be associated with a decrease in AKI incidence in patients undergoing unilateral partial nephrectomy.

## Materials and methods

### Ethical approval

The University of Texas MD Anderson Cancer Center Institutional Review Board approved this study (protocol #2020-0380), and a waiver of written consent was granted owing to its retrospective nature. This investigation is reported following the Strengthening the Reporting of Observational Studies in Epidemiology recommendations.

### Patients

Data from adult patients (≥ 18 years old) who underwent unilateral partial nephrectomy between April 1, 2016, and October 14, 2023, were extracted from electronical medical records and considered for analysis. Patients with a history of end-stage renal disease, absent preoperative blood pressure readings, or who underwent ineligible procedures (i.e., aborted procedure, conversion to radical nephrectomy, surgery on a horseshoe kidney) were excluded. Those who underwent reoperations within 3 days of the nephrectomy were also excluded. Patients were grouped according to whether dexmedetomidine was administered during the intraoperative period. All patients in the dexmedetomidine group received the drug intravenously (dosage, 0.2–0.7 µg/kg/h; initiated after induction of general anesthesia) as part of their anesthetic management. All patients receive general volatile anesthesia with opioid sparing strategies. In most patients, wound infiltration with a local anesthetic was done performed by the surgical team and minimization of nephrotoxic agents such as non-steroidal anti-inflammatory drugs was routinely done.

### Variables

Demographic variables were age at surgery, body mass index (BMI), sex, ethnicity, primary race, and American Society of Anesthesiologists (ASA) score. Procedure-related variables were anesthesia duration (minutes [min]), procedure duration (incision to procedure end, minutes), laterality (left or right), final surgical approach (minimally invasive or open), estimated parenchymal preservation (measured as an estimated percentage of kidney parenchyma), and total ischemia time (total clamp time, min).

Intraoperative hypotension (IOH) has been associated with AKI, and dexmedetomidine has been shown to cause IOH [25]. Absolute hypotension was defined as a mean arterial pressure less than 65 mm Hg. For patients who had baseline blood pressure (defined as the 6-month average of measured outpatient blood pressure) available, we calculated their relative hypotension. Relative hypotension was defined as a mean arterial pressure less than 0.8 times the baseline blood pressure. The time-weighted average for hypotension was defined as the proportion of time that patients were hypotensive during the procedure [26]. Intraoperative mean arterial pressure values were linearly interpolated for patients missing fewer than 20% of readings from the surgery. Avoidance of IOH and early treatment with vasopressors (i.e., ephedrine or phenylephrine) was practiced per our standard of care.

### Outcomes

The primary outcome of this study was the AKI incidence during the first 3 days after the operation. Development of an AKI was determined using the highest daily serum creatinine level in accordance with the Kidney Disease Improving Global Outcomes (KDIGO) definition. Secondary outcomes were the day of AKI onset, worst stage of AKI, day of the worst stage of AKI, length of hospital stay, and IOH.

### Statistical analysis

Categorical variables were compared using the chi-square test. Continuous variables were assessed for normality using the Shapiro–Wilk test. Non-parametric data were compared using the Mann–Whitney U test.

Propensity score matching (PSM) was conducted to adjust for potential bias. Patients in the dexmedetomidine and no dexmedetomidine groups were matched in a 1:1 ratio using a nearest-neighbor strategy. Patients' age, sex, body mass index, and ASA were included in the PSM *a priori* based on prior literature and biological relevance to AKI [27–29]. Covariates that were independent from dexmedetomidine administration and found to be significantly different between groups in the pre-matching analysis were also included in the PSM model.

Standardized mean differences were calculated for each covariate before and after PSM; covariates with a standardized mean difference greater than 10% were considered unbalanced during the matching process. A multivariable logistic regression model to evaluate the odds ratio for the primary outcome, the development of AKI.

A p value less than 0.05 was considered statistically significant. Statistical analysis was performed using SPSS (IBM) v. 25 software.

## Results

### All patients

**Figure 1.**
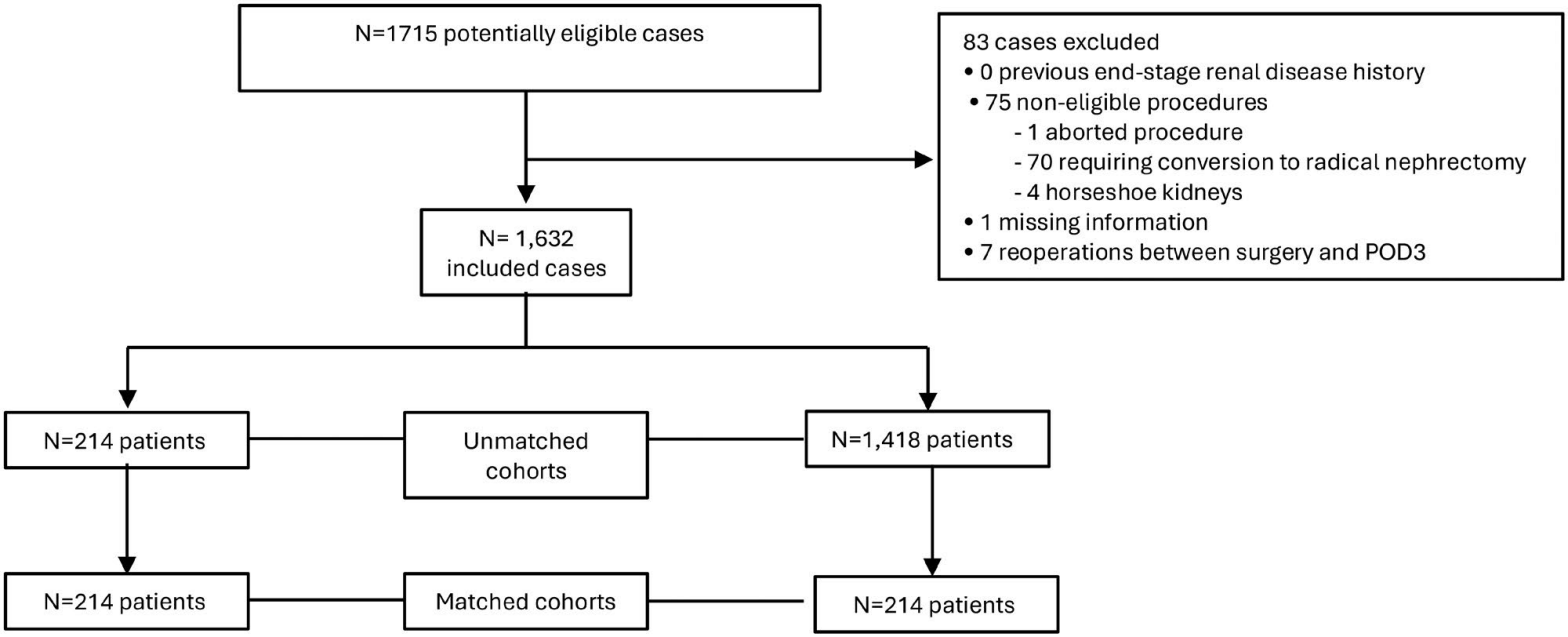
Flow diagram.

Of 1,715 screened patients, 1,632 met the eligibility criteria. Of these, 214 patients received dexmedetomidine and 1,418 did not (Figure 1). The median administered dose of dexmedetomidine was 63 µg (interquartile range [IQR]: 41–85 µg). Table 1 summarizes clinical characteristics for both the unmatched and matched cohorts.

**Table 1.** Pre-matching and post-matching perioperative according to dexmedetomidine intraoperative administration.

	Pre-matching cohort (*N* = 1,632)				Post-matching cohort (*N* = 428)		
	Dexmedetomidine (*n* = 214)	No Dexmedetomidine (*n* = 1418)	p-value	Dexmedetomidine (*n* = 214)	No Dexmedetomidine (*n* = 214)	SMD	p-value
Age at surgery	54.5 [46–66]	60 [50–67]	<0.001	55 [46–66]	56 [45–63]	0.065	
BMI	31.8 [27.9–35.9]	30.4 [26.9–34.6]	0.079	31.8 [27.9–35.9]	31.5 [27.8–35.3]	0.013	
Sex, n (%)			0.570			0.15	
Male	138 (64.5)	882 (62.2)		138 (64.5)	129 (60.3)		
Female	76 (35.5)	536 (37.8)		76 (35.5)	85 (39.7)		
Ethnicity, n (%)			0.568				0.630
Hispanic or Latino	48 (22.4)	291 (20.5)		48 (22.4)	51 (23.8)		
Not Hispanic or Latino	163 (76.2)	1088 (76.7)		163 (76.2)	157 (73.4)		
Declined to Answer	3 (1.4)	32 (2.3)		3 (1.4)	5 (2.3)		
Unknown	0 (0)	7 (0.5)		0 (0)	1 (0.5)		
Primary Race, n (%)			0.745				0.360
White or Caucasian	171 (79.9)	1069 (75.4)		171 (79.9)	146 (68.2)		
Black or AA	14 (6.5)	113 (8)		14 (6.5)	17 (7.9)		
Asian	5 (2.3)	40 (2.8)		5 (2.3)	7 (3.3)		
American Indian or Alaskan Native	3 (1.4)	12 (0.8)		3 (1.4)	3 (1.4)		
Native Hawaiian or Pacific Islander	0 (0)	1 (0.1)		–	–		
Other	18 (8.4)	165 (11.6)		18 (8.4)	40 (18.7)		
Unknown	0 (0)	3 (0.2)		–	–		
Declined to Answer	3 (1.4)	15 (1.1)		3 (1.4)	1 (0.5)		
ASA Score, n (%)			0.541			0.15	
I	0 (0)	2 (0.1)		0 (0)	1 (0.5)		
II	29 (13.6)	222 (15.7)		29 (13.6)	31 (14.5)		
III	177 (82.7)	1160 (81.8)		177 (82.7)	178 (83.2)		
IV	8 (3.7)	34 (2.4)		8 (3.7)	4 (1.9)		
Laterality, n (%)			0.412				
Left	98 (45.8)	692 (48.8)		98 (45.8)	98 (45.8)		1.000
Right	116 (54.2)	726 (51.2)		116 (54.2)	116 (54.2)		
Final Approach, n (%)			<0.001			0.441	
Minimally invasive	154 (72)	794 (56)		154 (72)	162 (75.7)		
Open	60 (28)	624 (44)		60 (28)	52 (24.3)		
Anesthesia duration (mins)	260 [213–306]	235 [157–285]	<0.001	260 [213–306]	268.5 [219–311]		0.439
Procedure duration (mins)	177.5 [132–220]	154.5 [89–205]	<0.001	177.5 [132–220]	179.5 [141–225]		0.923
Parenchymal preservation (%)	85 [80–90]	85 [80–90]	0.757	85 [80–90]	90 [80–90]		0.133
Total ischemia time (mins)	15.5 [11–20]	14 [10–18]	0.165	15.5 [10–20]	15 [10–20]		0.562
Ischemia time ≥ 15 min, n (%)			<0.001			0.499	
<15 min	106 (49.5)	901 (63.5)		101 (48.3)	113 (51.6)		
≥15 min	108 (50.5)	517 (36.5)		108 (51.7)	106 (48.4)		
**Length of stay in days, median [IQR]**	2.3 [1.5–3.3]	2.5 [2.1–3.5]	0.005	2.3 [1.5–3.3]	2.3 [1.6–3.3]		0.497

Continuous variables are shown as median [IQR]. AKI = Acute Kidney Injury; BMI = Body Mass Index; AA = African American; ASA = American Society of Anesthesiologists Physical Status Classification System.

### Unmatched cohort

Before matching, patients who received dexmedetomidine were statistically significantly younger (54.5, IQR: 46–66 years) than those who did not (60, IQR: 50–67 years; *p* < 0.001). A statistically significantly higher percentage of patients who received dexmedetomidine (72%) underwent minimally invasive surgeries compared with those who did not receive dexmedetomidine (56%; *p* < 0.001). Compared with those who did not receive dexmedetomidine, those who received dexmedetomidine had a statistically significantly longer median durations of anesthesia (235 min, IQR: 157–285 min, vs 260 min, IQR = 213–306 min; *p* < 0.001) and the procedure (154.5 min, IQR: 89–205 min, vs 177.5 min, IQR: 132–220 min; *p* < 0.001). The median total ischemia time did not differ significantly between the 2 groups (15.5 min, IQR: 11–20 min for those with dexmedetomidine vs. 14 min, IQR: 10–18 min for those without it; *p* = 0.165 However, the ischemia time exceeded 15 min in 50.5% of patients who received dexmedetomidine but in only 36.5% of patients who did not (*p* < 0.001). Other demographic and intraoperative variables were not significantly different between in groups (Table 1).

Intraoperative hemodynamic outcomes are presented in Table 2. Before matching, a slightly higher percentage of patients who received dexmedetomidine experienced absolute IOH compared with patients who did not receive dexmedetomidine, but the difference was not significant (74.1% vs 79.1%; *p* = 0.137). The percentages of patients with relative IOH were the same in the two groups (96.3% vs 96.4%; *p* = 0.921). Although the median duration of absolute IOH (3 min) and median absolute time-weighted average (1.5) were similar between groups, patients who received dexmedetomidine experienced slightly longer but not significantly different median relative IOH and had larger median relative time-weighted average than patients who did not.

**Table 2.** Intraoperative hemodynamic outcome variables.

	Pre-matching cohort			Post-matching cohort		
	Dexmedetomidine (*n* = 196)	No dexmedetomidine (*n* = 1213)	p-value	Dexmedetomidine (*n* = 196)	No dexmedetomidine (*n* = 190)	p-value
Absolute hypotension^§^ n (%)			0.137			0.823
Yes	155 (79.1)	899 (74.1)		155 (79.1)	152 (80)	
No	41 (20.9)	314 (25.9)		41 (20.9)	38 (20)	
Relative hypotension^†*5^ n (%)			0.921			0.566
Yes	189 (96.4)	1166 (96.3)		189 (96.4)	181 (95.3)	
No	7 (3.6)	45 (3.7)		7 (3.6)	9 (4.7)	
Absolute hypotension (min)	3 [1–8]	3 [0–9]	0.977	3.0 [1.0–8]	3 [1–10]	0.991
Relative hypotension (min)	44.5 [17.5–79]	36[13–72]	0.114	44.5 [17.5–79]	46 [20–94]	0.760
TWA-absolute hypotension (%)	1.5 [0.3–3.5]	1.5 [0–4.4]	0.573	1.5 [0.3–3.5]	1.5 [0.4–3.9]	0.919
TWA-relative hypotension (%)	19.3 [6.5–39.9]	17.5 [7.1–36.8]	0.509	19.3 [6.5–39.9]	19.7 [9.5–41.7]	0.919

Continuous data are shown as median [IQR]. BP = Blood Pressure, TWA = Time-Weighted Average.

§ Absolute hypotension = Mean arterial pressure < 65 mmHg, †Relative hypotension = Mean arterial pressure < 0.8 times the baseline BP, *^5^ = Relative hypotension pre-matching total *n* = 196 yes, *n* = 1211 no.

Table 3 displays renal outcomes in all groups. Overall, 431 (26.4%) patients developed AKI within the first 3 days after the operation. The univariate analysis showed that the rate of AKI among patients who received dexmedetomidine was higher (31.3%) than among those who did not (25.7%;), but the difference was not significant (*p* = 0.081). Most AKIs occurred on day 0 or 1 (91% among patients who received dexmedetomidine and 88.7% among those who did not; *p* = 0.679). The worst stage of AKI also most often occurred on day 0 or 1 (91% among patients who received dexmedetomidine and 87.6% among those who did not; *p* = 0.523). Most patients had a stage I AKI (97% among patients who received dexmedetomidine and 94% among those who did not); stage II AKI was slightly less frequent in patients who received dexmedetomidine (1.5% vs 4.7%; *p* = 0.489). One patient in each group developed AKI that required renal replacement therapy over postoperative days 2 and 3.

**Table 3.** Outcome variables for patients developing AKI.

	Pre-matching cohort			Post-matching cohort		
	Dexmedetomidine (*n* = 214)	No dexmedetomidine (*n* = 1418)	p-value	Dexmedetomidine (*n* = 214)	No dexmedetomidine (*n* = 214)	p-value
**AKI, n (%)**	**67 (31.3)**	**364 (25.7)**	**0.081**	**67 (31.3)**	**59 (27.6)**	**0.396**
**Day of AKI onset, n (%)**			0.679			0.546
POD0	27 (40.3)	121 (33.2)		27 (40.3)	19 (32.2)	
POD1	34 (50.7)	202 (55.5)		34 (50.7)	35 (59.3)	
POD2	6 (9)	36 (9.9)		6 (9)	4 (6.8)	
POD3	0 (0)	5 (1.4)		0 (0)	1 (1.7)	
**Worst stage of AKI, n (%)**			0.489			0.640
Stage 1	65 (97)	342 (94)		65 (97)	58 (98.3)	
Stage 2	1 (1.5)	17 (4.7)		1 (1.5)	1 (1.7)	
Stage 3	1 (1.5)	5 (1.4)		1 (1.5)	0 (0)	
**Day of worst AKI, n (%)**			0.523			0.662
POD0	26 (38.8)	109 (29.9)		26 (38.8)	19 (32.2)	
POD1	35 (52.2)	210 (57.7)		35 (52.2)	35 (59.3)	
POD2	6 (9)	40 (11)		6 (9)	4 (6.8)	
POD3	0 (0)	5 (1.4)		0 (0)	1 (1.7)	

AKI = Acute Kidney Injury, POD = Postoperative Day.

We also investigated the difference in the median administered dose of dexmedetomidine between patients who developed AKI and those who did not. The analysis showed median dexmedetomidine dose did not significantly differ between patients who had AKI (68.65 µg, IQR: 44.75–93 µg) and those who did not (63.36 µg, IQR: 40–0.75 µg; *p* = 0.968).

Patients who received dexmedetomidine had a significantly shorter median length of hospital stay (2.3 days, IQR:1.5–3.3 days) than those who did not (2.5 days, IQR: 2.1–3.5 days; *p* = 0.005; Table 1), although this difference is not clinically meaningful.

### Matched cohort

Age, BMI, sex, and ASA score were included in the PSM strategy because of their known biological effect on AKI development. Of the covariates that were independent of dexmedetomidine administration, final surgical approach and ischemia time were significantly different during unmatched bivariate analysis (both *p* < 0.001) between groups and thus included in the PSM strategy. As shown in Table 1, 214 patients were included in each group. Demographic variables, surgical approach, median anesthesia and procedure durations, and total ischemia time were well balanced after matching.

The univariate analysis showed no significant differences were found in the frequency of absolute and relative IOH and the median duration of relative IOH (Table 2). Patients who received dexmedetomidine had a higher, although not statistically significance, incidence of AKI during the first 3 days after the operation (31.3%) than patients who did not receive dexmedetomidine (27.6%; *p* = 0.376). A slightly higher percentage of patients who did not receive dexmedetomidine, compared with those who did receive dexmedetomidine, experienced their worst day of AKI during day 0 or 1. The severity of the AKI was mild (stage I) in almost all patients (Table 3).

The median length of stay was identical (2.3 days) between groups; no significant differences were observed between patients who received dexmedetomidine and those who did not (*p* = 0.497). Only 1 patient received renal replacement therapy in the matched cohort.

After adjusting for age, BMI, sex, ASA score, and surgical approach, the multivariate analysis demonstrated that dexmedetomidine administration during unilateral partial nephrectomy did not impact the development of AKI (odds ratio: 0.910, 95% CI: 0.585–1.142; *p* = 0.677) within 3 days of the surgery.

## Discussion

This study investigated the association between intraoperative dexmedetomidine and early postoperative AKI in patients undergoing unilateral partial nephrectomy. After accounting for demographic, intraoperative, and procedure-related variables, we found no association between intraoperative pre-ischemic dexmedetomidine administration and the rate and severity of AKI. These findings did not support our hypothesis. Our analysis is unique because it included variables such as clamp-related ischemia, IOH, and parenchymal preservation, which are known to contribute to AKI development following partial kidney resection [3,6,7,30–32].

Findings from murine models of renal ischemia/reperfusion injury have supported the use of dexmedetomidine to prevent AKI during nephrectomy [18–23,33]. However, clinical evidence is inconclusive. For instance, Su et al. did not find a significant reduction in the 7-day incidence of AKI as measured by serum creatinine levels following dexmedetomidine administration during laparoscopic radical nephrectomy [34]. Park et al. did not find a reduction in levels of plasma cytokine markers of AKI following continuous dexmedetomidine administration for living-donor kidney transplants [35]. Similarly, dexmedetomidine did not show strong renoprotective effects in a randomized controlled trial in patients undergoing liver resections [36]. A large randomized controlled trial in older patients who underwent major non-cardiac surgery also failed to show that dexmedetomidine reduced the incidence of AKI [37]. All those studies are consistent with our findings. However, Jiang et al. conducted a randomized controlled trial in 90 patients undergoing laparoscopic partial nephrectomy and reported a significant reduction in neutrophil gelatinase-associated lipocalin, a nephron epithelial injury marker, 2 and 6 h following bolus dexmedetomidine administration compared with a placebo (saline solution) [15]. In support of Jiang's work, a recent meta-analysis that included cardiac and non-cardiac surgeries showed that dexmedetomidine reduce AKI [24]. However, moderate heterogeneity was found in the meta-analysis. Interestingly, Jiang et al. administered a bolus of dexmedetomidine after clamping of the renal artery and found a protective effect. In our study, dexmedetomidine infusion typically was started without a bolus after the induction of general anesthesia, which could be considered a preischemic effect [15]. Other differences between our study results and Jiang et al.'s work included study design, AKI definition, patient populations, surgical settings, lack of information about warm ischemia in which dexmedetomidine was administered [15].

The median dose of dexmedetomidine in our study was 63 µg which is a low dose based on prior cardiac and non-cardiac studies [24]. While prior work has shown dexmedetomidine does not prevent AKI in a dose-dependent manner during cardiac surgery, it possible to speculate that a higher dose may have shown protective effects [38].

Our study contains several limitations in addition to those inherent in its retrospective nature. First, the dexmedetomidine administration regimens were determined by each anesthesiologist, which may have introduced variability into the data. Second, because of our small sample size, we did not compare whether dexmedetomidine had differing effects on AKI based on surgical approach. Third, urine output data were unavailable, limiting the AKI assessment to changes in serum creatinine level. Also, function of the non-operated kidney was not available in the electronic medical records. Fourth, the study focused solely on short-term outcomes, excluding AKI that persisted for more than 3 days after the operation or chronic kidney disease. However, it has been shown that patients who do not recover their renal function within 3 days after nephrectomy have an increased risk of chronic kidney disease, so our findings still may be relevant for patients with chronic kidney disease [6]. Finally, we did not include other drugs (i.e., antibiotics) that could have affected postoperative AKI onset in the analysis nor the impact of vasopressor use during surgery [39].

In conclusion, intraoperative dexmedetomidine was not significantly associated with decreased development of postoperative AKI after unilateral partial nephrectomy. Future studies are needed to elucidate the effect of pre- and post-conditioning dexmedetomidine on renal outcomes in this setting.

## Data Availability

Any requested statistical data not in violation of the HIPAA statute is available to any correspondents without charge upon request.
